# SIRT3 Mediates Coordination Between Energy Metabolism and SOD Activity in Melatonin-Enhanced Boar Sperm Motility

**DOI:** 10.3390/cells14201633

**Published:** 2025-10-20

**Authors:** Naisheng Lu, Hulong Lei, Xueyuan Jiang, Peng Jia, Bushe Li, Dong Xia

**Affiliations:** 1 Key Laboratory of Livestock and Poultry Resources (Pig) Evaluation and Utilization of Ministry of Agriculture and Rural Affairs, Institute of Animal Husbandry and Veterinary Medicine, Shanghai Academy of Agricultural Sciences, Shanghai 201106, China; leihulong@saas.sh.cn (H.L.); jiangxueyuan@saas.sh.cn (X.J.); jiapeng@saas.sh.cn (P.J.); 2 Shanghai Engineering Research Center of Breeding Pig, Shanghai 201302, China; fordcc@163.com

**Keywords:** melatonin, boar sperm, SIRT3, mitochondrial deacetylation, energy metabolism, SOD activity, ATP production

## Abstract

**Highlights:**

**What are the main findings?**

- SIRT3 mediates the coordinated enhancement of energy metabolism and SOD activity by MLT in boar sperm.
- This dual regulation enhances metabolic flux and ATP synthesis, thereby boosting sperm motility.

**What are the implications of the main findings?**

- Targeting SIRT3 emerges as a promising strategy to ameliorate boar fertility.
- The MLT-SIRT3 axis offers novel mechanistic insights into human male infertility.

**Abstract:**

Previous studies have demonstrated that melatonin (MLT) enhances boar sperm motility by modulating energy metabolism status, yet the underlying mechanisms remain incompletely understood. This study aims to investigate whether sirtuin 3 (SIRT3), a key mitochondrial deacetylase, mediates MLT’s effects. Herein, the semen of six Landrace boars (16–18 months of age) was treated with 1.0 μM MLT with/without the SIRT3 inhibitor 3-TYP, preserved at 17 °C for 3 days, and subsequently maintained at 37 °C for a duration of 10 min. We demonstrated that MLT upregulated SIRT3 protein expression and reduced the acetylation level in mitochondrial proteins. MLT significantly increased glucose uptake and suppressed lactate release in the sperm, while elevating levels of pyruvate and acetyl-CoA, the substrates of pyruvate dehydrogenase (PDH) and the tricarboxylic acid (TCA) cycle, respectively, and the protein expression of PDH, indicating enhanced metabolic flux. Notably, inhibition of SIRT3 reversed MLT’s effects: it blocked the increases in SIRT3 expression, glucose consumption, PDH expression, complex I activity, ATP content, and superoxide dismutase (SOD) activity, and prevented the decreases in the levels of acetylation and lactate, as well as pyruvate kinase (PK) activity, confirming the essential role of SIRT3. Functionally, the MLT-induced improvements in sperm motility parameters (total, progressive, fast motility, immotile) were also reversed by 3-TYP. Collectively, these findings demonstrate that the SIRT3-mediated pathway is essential for MLT to enhance boar sperm energy metabolism and antioxidant defense, thereby increasing ATP production and enhancing sperm motility. Targeting SIRT3 represents a promising therapeutic strategy for improving boar fertility and may also provide insights for research into human male infertility.

## 1. Introduction

During in vitro liquid preservation, boar sperm experience a rapid decline in motility within 2–3 days, even at optimal storage temperatures (15–20 °C) [1]. This decline is attributed not only to ATP depletion and oxidative damage from basal metabolic maintenance [2], but more critically by insufficient ATP synthesis capacity during sperm motion (motility activation); this ultimately impairs fertilizing capacity and reduces fertility [3]. Therefore, it is crucial for sperm to have an optimal energy metabolism that matches the varying microenvironments they encounter.

Mammalian spermatozoa are defined by their structural specialization: the head is characterized by the presence of the nucleus and acrosomal, and the neck and tail contain mitochondria. Despite this structural specialization, the execution of their critical functions, such as capacitation, hyperactivation, motility, and acrosome reaction, is driven by ATP-derived energy [4]. This ATP is primarily generated by the contribution of two principal metabolic routes: glycolysis, localized in the head and the tail, and oxidative phosphorylation (OXPHOS) within the mitochondria of the midpiece [4]. In pig spermatozoa, glycolysis serves as the dominant route for glucose metabolism [5], but aerobic metabolism is also required [6]. Spermatozoa can survive purely on glycolytic energy, yet they rely on OXPHOS for differentiation and maturation [7]. Importantly, they can modulate the functions of these two pathways to satisfy their energy demands based on different conditions and fertilization stages [8], indicating a cooperation and competition in ATP production between glycolysis and OXPHOS [9]. This has led to a long-standing scientific debate (sperm energy debate) regarding their relative contributions to ATP generation. However, current research reveals that sperm from the majority of mammalian species utilize a flexible metabolic approach to sustain motility in response to physiological conditions [10,11]. Various metabolic pathways—including glycolysis, the tricarboxylic acid cycle (TCA), OXPHOS, etc.—appear to synergize by utilizing different external and internal substrates for effective ATP production [12,13]. Nevertheless, the specific regulatory mechanisms governing this metabolic coordination in boar sperm under varying environmental conditions remain unclear.

Sirtuins, a conserved family of nicotinamide adenine dinucleotide (NAD^+^)-dependent deacetylases, have emerged as crucial regulators of cellular energy balance and the redox state [14]. Of the seven sirtuin proteins (SIRT1–7) found in mammals, SIRT3 is predominantly localized in mitochondria, where it can regulate the enzymatic activity of the mitochondrial proteins involved in fatty acid oxidation and the TCA cycle [15]. SIRT3 can also activate pyruvate dehydrogenase (PDH) by reducing the acetylation levels of mitochondrial proteins and induce a switch in cardiac energy substrate utilization from fatty acid β-oxidation to glucose oxidation [16]; additionally, it enhances electron transport through deacetylation of complexes I and II [15]. SIRT3 knockout can cause hyperacetylation of mitochondrial proteins and multiple components of complex I, resulting in a reduction in the activity of complex I and a decrease in ATP synthesis [15,17]. Beyond its metabolic role, SIRT3 exerts critical antioxidant effects by deacetylating and activating superoxide dismutase (SOD2) to protect porcine sperm from damage and apoptosis caused by stress [18]. These findings establish SIRT3 as a central regulator of energy and redox homeostasis. However, the role of SIRT3 in coordinating energy metabolism and antioxidants in boar sperm is still largely unexplored.

Melatonin (MLT) is a neurohormone that has been demonstrated to be present in multiple organs, including the pineal gland and retina, among others; it is also detected in the testes of mice [19] and the seminal plasma of pigs [20]. Additionally, MLT-related enzymes are expressed in pig testicular tissue [21]. MLT is known to protect boar sperm and improve sperm quality by maintaining acrosomal and plasma membrane integrity, reducing reactive oxygen species (ROS), superoxide, and apoptosis, and enhancing mitochondrial function and antioxidant enzyme activity [22,23]. Notably, mitochondria have a comparatively high MLT level and are a primary site of MLT action [24]; emerging evidence links MLT to the regulation of glucose homeostasis and energy metabolism [25], which in turn alleviates impairment of Sertoli cells [26]. Our previous study demonstrated that treating boar sperm with MLT on the third day of storage, followed by incubation at 37 °C, enhanced OXPHOS and SOD activity, weakened glycolysis, increased ATP synthesis, and significantly increased sperm motility [27]. This strongly suggests that MLT affects the coordination between energy metabolism and antioxidant capacity. However, the specific regulatory mechanisms and signaling pathways involved remain unknown. We thus hypothesize that MLT’s beneficial effects are mediated through the activation of SIRT3, forming a functional axis that coordinates energy metabolism and antioxidant activity.

To verify this, we treated boar semen with 1.0 μM MLT (with or without the specific SIRT3 antagonist 3-TYP) during storage at 17 °C. After three days, samples were incubated at 37 °C for 10 min, followed by systematic analysis of the following: SIRT3 protein level; mitochondrial protein acetylation level; changes in metabolic flux; key enzyme activities or expression in the glycolytic or TCA cycles; OXPHOS and SOD activity; and ATP content, as well as sperm parameters. We demonstrated that MLT enhances boar sperm energy metabolism and antioxidant defense through SIRT3-mediated mechanisms, promoting OXPHOS while reducing glycolysis activity and boosting SOD activity. This metabolic coordination—likely facilitated by SIRT3-dependent deacetylation—contributes to the improvement of ATP generation and sperm parameters.

## 2. Materials and Methods

All experimental procedures in this study were performed in strict accordance with the Laboratory animal-Guideline for Ethical Review of Animal Welfare of China and received approval from the Laboratory Animal Ethics Committee of Shanghai Academy of Agricultural Sciences (Approval code: SAASPZ0521008).

### 2.1. Samples and Treatments

Six healthy and sexually mature Landrace boars (average age: 16.9 ± 0.4 months, range: 16–18 months) were used in this study, with a total of 18 ejaculates (3 per boar) collected. All boars were housed in individual pens in an environmentally controlled building (18–22 °C) and fed a standard balanced diet at the Shanghai Engineering Research Center of Breeding Pig (Shanghai, China). Artificial insemination with the preserved liquid semen of these boars proved they were fertile.

During autumn, sperm-rich fractions were collected once per week by the gloved hand technique and immediately placed in a water bath at 38 °C. After collection, the sperm motility and concentration were assessed using computer-assisted sperm analysis (CASA) (AndroVision, Minitüb GmbH, Tiefenbach, Germany). Only ejaculates meeting strict quality criteria (≥80% motility and total sperm count > 10.0 × 10^9^ per ejaculate) were employed in the study. Following sample collection, each ejaculate was individually processed to achieve a final sperm concentration of 2.0 × 10^7^ cells/mL using the commercial semen diluent extender (ZENOLONG, ZENOAQ Co., Ltd., Tokyo, Japan).

To clarify the role of MLT (M5250, Sigma, Darmstadt, Germany) in glucose metabolism (*n* = 6), TCA key enzymes, and SIRT3 protein level (*n* = 3), etc., in sperm, every sample of diluted semen was subjected to either vehicle control (VC) or 1.0 μM MLT, and then stored away from light in an incubator set at 17 °C for 3 days. To test and verify the mechanism of MLT on sperm energy metabolism (*n* = 3–4), each diluted semen sample was treated with VC, 1.0 μM MLT, 10.0 μM 3-TYP (SIRT3 inhibitor, GC19013, GLP Bio Technology LLC., Montclair, CA, USA), 1.0 μM MLT + 10.0 μM 3-TYP and kept away from light in the incubator at 17 °C for 3 days. Each of the above-mentioned treatment groups was divided into 20 parallel sperm samples, with each sample having a volume of 1 mL in a tube. The concentrations of MLT (1.0 μM) and 3-TYP (10.0 μM) were carefully chosen based on the literature [27,28]. Dimethyl sulfoxide (DMSO, D2650, Sigma, Darmstadt, Germany) was utilized to dissolve MLT and 3-TYP, with all experimental groups containing a final concentration of 0.1% DMSO.

On the third day, 19 tubes of 1 mL diluted semen from each treatment were incubated in a 37 °C water bath for 10 min, and the sperm pellets were harvested by centrifugation at 6000× *g*, 4 °C, 5 min, and then preserved at −80 °C for subsequent indicator detection, with sampling as needed. Meanwhile, the semen supernatants were collected for determination of glucose consumption and lactate secretion. Meanwhile, sperm parameters were analyzed in an aliquot using a CASA (AndroVision, Minitüb GmbHM, Tiefenbach, Germany) equipped with a 37 °C heated stage. Briefly, 10 µL of each semen sample was placed on a preheated (37 °C) counting chamber (ML-CASA10-4, 10 µm, Nanning Songjiang Tianlun Biotechnology Co., Ltd., Nanning, Guangxi, China) followed by an analysis of 4 microscopic fields per subsample to count at least 500 spermatozoa.

### 2.2. Mitochondria Isolation

Mitochondria were isolated from sperm using a Mitochondria Isolation Kit (C3601, Beyotime, Shanghai, China) as previously described [29]. Briefly, the sperm pellet was washed twice with ice-cold PBS, re-suspended in lysis buffer for 15 min, and then homogenized under ice-cold conditions and sequentially centrifuged. After initial clarification at 600× *g* for 10 min (4 °C) to remove debris and nuclei, the supernatants were subjected to high-speed centrifugation at 11,000× *g* for an additional 10 min at 4 °C. The mitochondria were collected in the sediments, which were observed under a microscope without the midpieces or tails of the sperm. The resulting mitochondrial pellets were re-suspended in mitochondria lysis solution. The supernatants and mitochondrial fractions of the sperm were stored at −80 °C until analysis.

### 2.3. Glucose, Lactate, and Pyruvate Assay

The glucose and lactate concentrations of the semen supernatant, and the pyruvate content of the sperm, were determined by a colorimetric method using a Multiskan Spectrum (SpectraMax^®^ ABS, Molecular Devices LLC., Sunnyvale, CA, USA). The assays were conducted following the manufacturer’s protocols provided for the following commercial kits: glucose (A154-2-1), lactate (A019-2-1), and pyruvate (A081-1-1) (Jiancheng, Nanjing, China). Glucose consumption was calculated as the difference between the glucose concentration in the semen extender on day 0 and that measured in the seminal supernatant after 3 days of storage. Lactate secretion was represented by its absolute concentration accumulated in the seminal supernatant by day 3. All results were referenced against protein concentrations, which were determined for total and mitochondrial sperm samples using the Pierce™ BCA Protein Assay Kit (23225, Thermo Fisher Scientific Inc., Waltham, MA, USA).

### 2.4. Acetyl-CoA Measurement

The acetyl-CoA of the sperm mitochondrial fraction was measured according to the manufacturer’s protocol with an acetyl-CoA assay Kit (ab87546, Abcam, Cambridge, UK). Briefly, after deproteinization using perchloric acid, CoASH Quencher and Quencher remover were added into the sample to correct the background generated by free CoASH and succ-CoA. The sample was then diluted with the reaction mix, and fluorescence was measured using a fluorescence plate reader (SpectraMax^®^ iD5, Molecular Devices LLC., Sunnyvale, CA, USA) and the following settings: 535 nm excitation and 587 nm emission wavelengths. The acetyl-CoA standard curve was formed in the range of 0–100 pM and the correlation coefficient was 0.990 or higher. The results were normalized to the mitochondrial protein concentrations of the samples.

### 2.5. NAD^+^/NADH Ratio Assay

NAD^+^ and NADH were examined by an assay kit from Beyotime Biotechnology (S0175, Shanghai, China) using the WST-8 method. The mitochondrial fractions of sperm were lysed using a special extraction solution. After centrifugation, the supernatant was used as the sample for testing. The sample was added directly to a 96-well plate for total NAD^+^/NADH detection (NAD total). After being heated in a 60 °C water bath for 30 min to remove NAD^+^, the sample was used to detect NADH content. Then, an alcohol dehydrogenase solution was added to the sample, which was then incubated in the dark at 37 °C for 10 min, followed by addition of color developing solution and incubation in the dark at 37 °C for 30 min. The NADH content was determined by Multiskan Spectrum (SpectraMax^®^ ABS, Molecular Devices LLC., Sunnyvale, CA, USA). NAD^+^ content = NAD total − NADH content.

### 2.6. Measurement of Mitochondria Membrane Potential (MMP)

Sperm MMP was assessed using the JC-1 Detection Kit (C2003S, Beyotime, Shanghai, China), following the manufacturer’s instructions. The JC-1 exhibits membrane potential-dependent spectral properties: in energized mitochondria, it forms aggregates with excitation/emission maxima at 525/590 nm, while in depolarized mitochondria, it remains monomers with excitation/emission maxima at 490/530 nm. Briefly, 100 μL of sperm suspension (1.0 × 10^6^ cells/mL) were stained with 500 μL of JC-1 working solution at 37 °C for 20 min under light-protected conditions. Following centrifugation at 600× *g* for 3 min (4 °C), the samples were subjected to two washes and finally re-suspended in JC-1 buffer in ice water. The fluorescence intensity of aggregates and monomer were detected with a microplate reader (SpectraMax^®^ iD5, Molecular Devices LLC., Sunnyvale, CA, USA) to calculate the ratio of aggregates to monomer.

### 2.7. Mitochondrial Complex I Activity Measurement

Mitochondrial complex I activity was quantified using a spectrophotometric assay kit (700930, Cayman Chemical Company, Ann Arbor, MI, USA) performed on a Multiskan Spectrum microplate reader (SpectraMax^®^ ABS, Molecular Devices LLC., Sunnyvale, CA, USA). Results were expressed as absorbance changes per minute per milligram of protein, following the manufacturer’s protocol.

### 2.8. The Activities of Pyruvate Kinase (PK) and SOD Measurement

The sperm pellet was thoroughly lysed in an ice-water bath for 10 min using PBS, a process that yielded a homogenate. Subsequent centrifugation at 600× *g* and 4 °C for 10 min allowed for the recovery of the supernatant. Following this, the enzymatic activity levels of pyruvate kinase (PK) and SOD in the supernatant were assayed via colorimetric techniques, with a UV spectrophotometric device (EMC-18PC-UV, EMCLAB Instruments GmbH, Duisburg, Germany) and a Multiskan Spectrum (SpectraMax^®^ ABS, Molecular Devices LLC., Sunnyvale, CA, USA), respectively. The assays were performed according to the manufacturer’s instructions for the following commercial kits: PK (A076-1-1), SOD (A001-3-2) (Jiancheng, Nanjing, China). All results were referenced against the protein concentrations of the samples.

### 2.9. ATP Content Measurement

The measurement of sperm ATP content was performed with an Enhanced ATP Assay Kit (S0027, Beyotime, Shanghai, China) as previously described, with some modifications [30]. Briefly, the collected sperm pellet was fully lysed (homogenate) with 500 μL ATP lysis buffer incubated in an ice bath for 10 min, and then the supernatant was collected by 10 min centrifugation at 12,000× *g* at 4 °C. Afterward, 20 μL supernatant was mixed with 100 μL of luciferase reagent, and the fluorescence intensity was determined by a microplate reader (SpectraMax^®^ iD5, Molecular Devices LLC., Sunnyvale, CA, USA). The results were referenced against the protein concentrations of the samples.

### 2.10. Western Blot Assay

After isolating the sperm mitochondrial fraction, proteins were thoroughly extracted using a mitochondrial lysis buffer containing 1.0 mM phenylmethanesulfonyl fluoride (PMSF), and the protein concentration was determined via the Pierce™ BCA Protein Assay Kit (23225, Thermo Fisher Scientific Inc., Waltham, MA, USA). Subsequently, 15 micrograms of mitochondria protein extract was subjected to electrophoresis with 10% SDS-PAGE gels. After separation, the samples were transferred to polyvinylidene fluoride (PVDF) (IPVH00010, Merck KGaA, Darmstadt, Germany) membranes and blocked with 5% skim milk solution. The following antibodies were used: mouse anti-cytochrome-c oxidase subunit IV (COX IV) (1:500, Santa, sc-376731), mouse anti-SIRT3 (1:1000, Santa, sc-365175), rabbit anti-acetylated lysine (1:1000, CST, 9441s), rabbit anti-PDH (1:1000, CST, 3205s), rabbit anti-citrate synthase (CS) (1:1000, Bioss, bs-17709R), and mouse anti-isocitrate dehydrogenase (IDH 1/2) (1:500, Santa, sc-373816). Information on the cross-reactivity of each antibody is provided in the Supplementary Materials File, Table S1. Membranes were incubated with primary antibodies at 4 °C overnight, followed by corresponding species-matched secondary antibodies (1:5000, Bioss). Protein signals were detected using the Tanon-5200 Chemiluminescent Imaging System (Tanon Science & Technology Co., Ltd., Shanghai, China), and the intensities were quantified using ImageJ software (ver. 1.51j8, Bethesda, MD, USA). The COX IV, acting as a loading control, was utilized to normalize the expression levels of the target proteins, as previously reported [31].

### 2.11. Statistical Analysis

The experimental data were organized and graphically presented using Microsoft Excel (Office Professional Plus 2013). All statistical analyses were conducted in SPSS 18.0 for Windows, employing paired-samples t-tests and one-way ANOVA to evaluate group differences, with statistical significance set at *p* < 0.05 for all determinations. Data were presented as means ± SEM throughout the study.

## 3. Results

### 3.1. The Levels of SIRT3 and Acetylation of Mitochondrial Proteins

**Figure 1:**
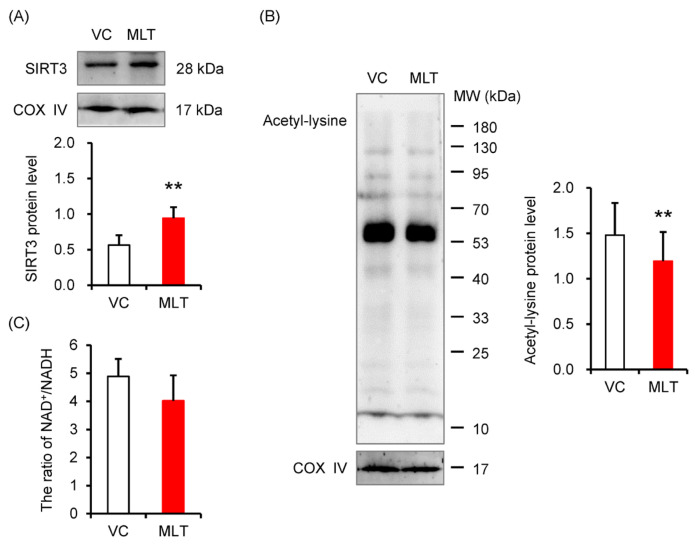
The levels of SIRT3 and acetylation of mitochondrial proteins in boar sperm. (**A**) SIRT3 protein level, (**B**) The acetylation level of mitochondrial protein, (**C**) NAD^+^/NADH ratio. VC, vehicle control (0.1% DMSO); MLT, melatonin. Data were expressed as the mean ± SEM ((**A**)/(**B**), *n* = 3; (**C**), *n* = 6), ** shows a significant change between the MLT and VC groups, ** *p* < 0.01.

In the mitochondrial protein fraction, the results showed that MLT significantly increased SIRT3 protein level (*p* = 0.002, Figure 1A) and concurrently decreased acetylation level (*p* = 0.018, Figure 1B) in boar sperm. To determine if this activation was driven by an increased availability of its essential cofactor, we measured the NAD^+^/NADH ratio. We found that the NAD^+^/NADH ratio remained unchanged between the VC and MLT groups (Figure 1C).

### 3.2. MLT Enhanced the Metabolic Flux in Boar Sperm

**Figure 2:**
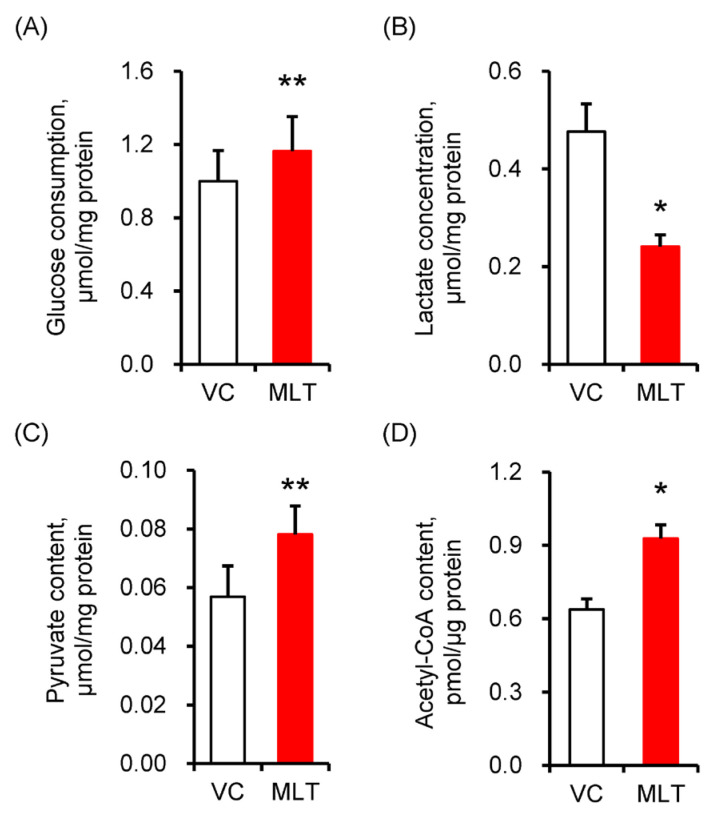
The changes in key metabolic flux in boar sperm. (**A**) Glucose consumption, (**B**) lactic acid concentration, (**C**) pyruvate content, (**D**) acetyl-CoA content. VC, vehicle control (0.1% DMSO); MLT, melatonin. Data were expressed as the mean ± SEM (*n* = 6), *^,^ ** shows a significant change between the MLT and VC groups, * *p* < 0.05, ** *p* < 0.01.

As shown in Figure 2, compared to the VC treatment, the MLT-treated samples showed an increase in the glucose consumption of the sperm (*p* = 0.006, Figure 2A), and a decrease in the lactate secretion (*p* = 0.020, Figure 2B) of the semen supernatant. Meanwhile, the MLT-treated sperm had a higher pyruvate content (the substrate of PDH) and acetyl-CoA content (the substrate of TCA cycle) compared to the VC-treated sperm (*p* = 0.005, 0.011, Figure 2C,D).

### 3.3. MLT Modulated the Protein Expression of Key Metabolic Enzymes

**Figure 3:**
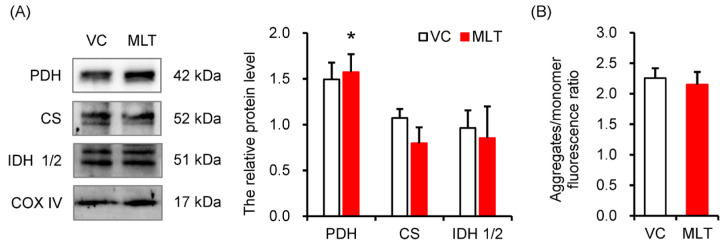
The protein levels of key metabolic enzymes and mitochondria membrane potential (MMP) of boar sperm. (**A**) The protein levels of pyruvate dehydrogenase (PDH), citrate synthase (CS), and isocitrate dehydrogenase 1/2 (IDH 1/2), (**B**) MMP, aggregates/monomer ratio. VC, vehicle control (0.1% DMSO); MLT, melatonin. Data were expressed as the mean ± SEM ((**A**), *n* = 3; (**B**), *n* = 6), * shows a significant change between the MLT and VC groups, * *p* < 0.05.

We next assessed the protein expression of key enzymes in the pivotal PDH and TCA cycle pathways. The MLT-treated sperm showed an increase in PDH protein level (*p* = 0.036), but no effect on the protein levels of CS and IDH 1/2 (Figure 3A). Additionally, there was no significant difference in MMP (aggregates/monomer) between the two groups (Figure 3B).

### 3.4. 3-TYP Abolished the MLT-Increased SIRT3 Level and MLT-Suppressed Acetylation Level

**Figure 4:**
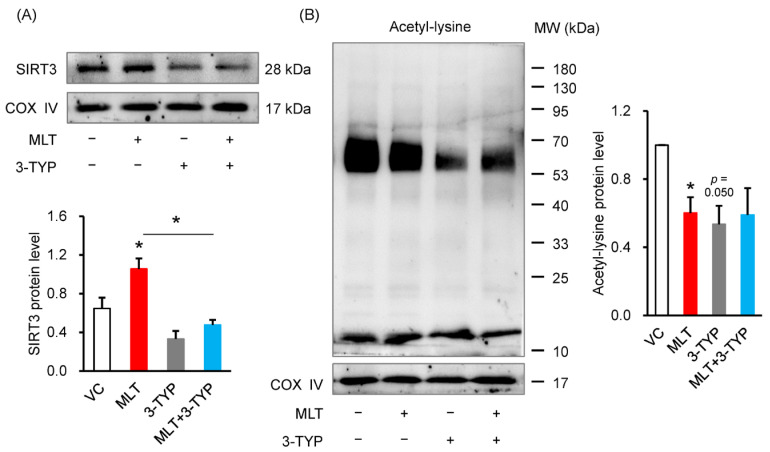
Influence of 3-TYP on the levels of SIRT3 and acetylation of mitochondrial proteins in boar sperm under MLT treatment. (**A**) SIRT3 protein level, (**B**) The acetylation level of mitochondrial protein. VC, vehicle control (0.1% DMSO); MLT, melatonin; 3-TYP, SIRT3 inhibitor. Data were expressed as the mean ± SEM (*n* = 3), * shows a significant change between the groups of MLT and VC, and MLT and MLT + 3-TYP, * *p* < 0.05.

We used 3-TYP to establish that MLT exerted its regulatory effect on SIRT3 and acetylation levels in the mitochondrial protein fraction. Exposure to MLT + 3-TYP reversed the stimulative effect of MLT on the SIRT3 level (*p* = 0.012, 0.042, Figure 4A) and the inhibitory effect of MLT on mitochondrial protein acetylation (*p* = 0.049, Figure 4B).

### 3.5. Inhibition of the SIRT3 Signaling Pathway Reversed the MLT-Induced ATP Synthesis of Sperm

**Figure 5:**
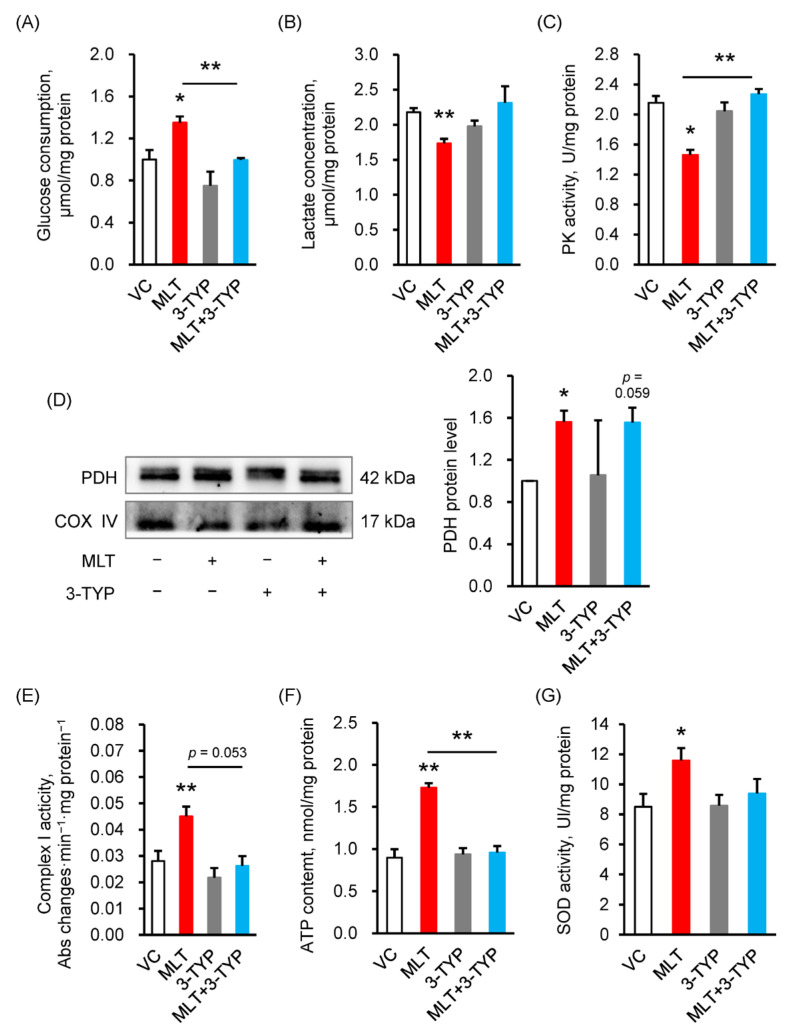
The SIRT3 signaling pathway mediates MLT’s regulation of energy metabolism, ATP synthesis and antioxidant capacity in boar sperm. (**A**) Glucose consumption, (**B**) lactic acid concentration, (C) pyruvate kinase (PK) activity, (**D**) Pyruvate dehydrogenase (PDH) protein level, (**E**) Complex I activity, (**F**) ATP content, (**G**) superoxide dismutase (SOD) activity. VC, vehicle control (0.1% DMSO); MLT, melatonin; 3-TYP, SIRT3 inhibitor. Data were expressed as the mean ± SEM ((**A**–**C**)/(**E**–**G**), *n* = 4; (**D**), *n* = 3), *^,^ ** shows a significant change between the groups of MLT and VC, and MLT and MLT + 3-TYP, * *p* < 0.05, ** *p* < 0.01.

As shown in Figure 5A, the MLT-induced increase in glucose consumption (*p* = 0.031) was significantly reversed by 3-TYP in boar sperm of MLT + 3-TYP treatment group (*p* = 0.009, MLT + 3-TYP vs. MLT). Similarly, exposure to MLT + 3-TYP reversed the MLT-induced decreases in lactate concentration (*p* = 0.004, Figure 5B). With respect to the energy metabolism pathways of glycolysis and OXPHOS, exposure to MLT + 3-TYP significantly reversed the MLT-induced decreases in PK activity (*p* = 0.016, 0.006, Figure 5C), and MLT-induced increases in PDH protein level (*p* = 0.035, Figure 5D) and complex I activity (*p* = 0.001, 0.053, Figure 5E). Subsequently, MLT-induced increases in ATP content (*p* = 0.007) were significantly reversed by 3-TYP in the boar sperm of the MLT + 3-TYP treatment group (*p* = 0.006, MLT + 3-TYP vs. MLT, Figure 5F). Finally, the SOD activity, a downstream effect of the SIRT3 pathway which is essential for production of ATP and antioxidant defense, was also measured. Exposure to MLT + 3-TYP significantly attenuated MLT-induced upregulation of SOD activity in boar sperm (*p* = 0.023, Figure 5G).

### 3.6. Inhibition of the SIRT3 Signaling Pathway Reversed the MLT-Induced Sperm Parameters

As shown in Table 1, compared to the VC treatment group, the MLT treatment group significantly increased the sperm motility (*p* = 0.000), progressive motility (*p* = 0.011), fast motility (*p* = 0.025), and decreased the immotile (*p* = 0.000). Single addition of 3-TYP did not affect the sperm parameters, and exposure to MLT + 3-TYP significantly reversed the MLT-induced motility (*p* = 0.001), fast motility (*p* = 0.027), and immotile (*p* = 0.001).

## 4. Discussion

**Figure 6:**
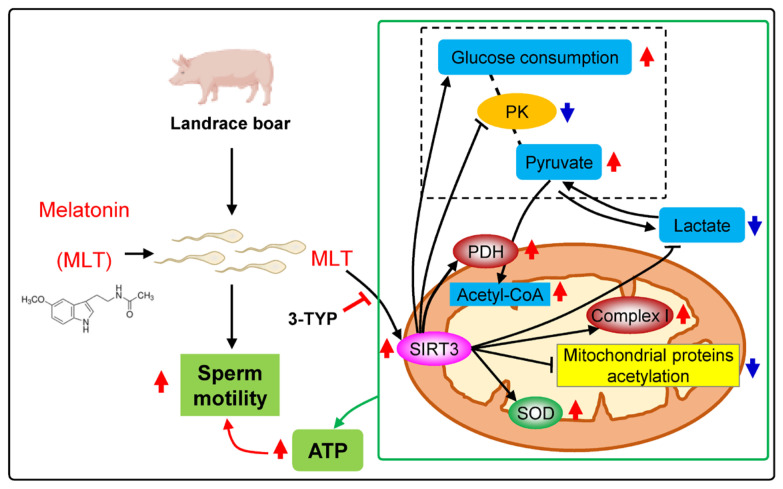
Schematic figure of the signaling pathway by which SIRT3 mediates melatonin (MLT) to coordinate energy metabolism and superoxide dismutase (SOD) activity for enhancing sperm motility. Red arrows indicate an increase, and blue arrows indicate a decrease.

This study identified the activation of the mitochondrial deacetylase SIRT3 as the central mechanism through which MLT enhances boar sperm motility. As delineated in Figure 6, MLT-upregulated SIRT3 reduces mitochondrial protein acetylation, enhances the activity and expression of key metabolic enzymes, and boosts SOD activity. These synergistic effects collectively improve metabolic efficiency and ATP synthesis.

A large number of animal studies have highlighted a role for MLT in the regulation of both glucose metabolism and energy balance [25]. Brydon et al. (2001) demonstrated that MLT decreased glucose transporter 4 (GLUT4) protein level and glucose uptake in adipocytes [32], while Rocha et al. (2014) revealed that MLT increased GLUT1 level yet suppressed lactate dehydrogenase (LDH) activity in Sertoli cells, thereby increasing glucose consumption but decreasing lactate production [33]. The authors speculated that MLT increased TCA cycle activity by promoting pyruvate entry. Consistently, studies on frozen–thawed ram sperm further demonstrated that MLT treatment led to decreased lactate concentrations, improved acetyl-CoA concentration, ATP production, and sperm motility [34]. Together, these findings demonstrate that MLT cell-specifically regulates glucose metabolism. In light of this, we evaluated MLT in boar sperm. Aligning with prior reports, MLT treatment significantly increased glucose consumption and decreased lactate secretion. This phenotype indicates a reprogramming of the pyruvate metabolism, likely involving the sperm-specific lactate dehydrogenase (LDH-C4), which is expressed in the cytoplasm and mitochondria of boar sperm, facilitates the conversion between lactate and pyruvate, and regulates ATP production [35]. The observed decrease in lactate secretion in this study suggests that MLT promotes the channeling of pyruvate, via PDH [36], into the TCA cycle for OXPHOS rather than its reduction to lactate.

PDH is essential for mitochondrial pyruvate influx and acetyl-CoA biosynthesis, with its knockout severely reducing acetyl-CoA and ATP levels in murine livers and tumors [37]. Additionally, pyruvate and acetyl-CoA are critical for mitochondrial ATP generation and in multiple biosynthetic pathways linked to the TCA cycle [38], as well as energy obtainment of boar spermatozoa [39,40]. Supporting this essential role, pyruvate treatment elevated intracellular ATP levels by 56%, enhanced progressive motility by 21%, and boosted glycolytic flux by 50% in human spermatozoa [41]. Accordingly, here, MLT also increased both pyruvate and acetyl-CoA contents in boar sperm, along with increasing PDH expression. Based on the above, this indicates that MLT enhances the metabolic flux of boar sperm. Critically, the SIRT3 inhibitor 3-TYP reversed the increased glucose consumption, decreased lactate production, and elevated the PDH level and ATP production induced by MLT. This confirms that SIRT3 drives the metabolic shift toward preferential glucose utilization for mitochondrial OXPHOS over glycolytic lactate generation—an adaptation that enables more efficient ATP production, thereby supporting sperm motility.

Proper mitochondrial function is crucial for sperm motility and function. Consequently, their optimal function underpins sperm quality, whereas any impairment leads to declined motility, concentration, and viability, ultimately contributing to male infertility [42,43]. Protein acetylation has emerged as a crucial regulatory mechanism in this context, particularly affecting enzymatic activity, metabolic pathways and energy production [44,45]. This modification is highly prevalent in mitochondria, where over one-third of mitochondria proteins—most involved in energy metabolism—undergo lysine acetylation [46,47]. While both enzymatic and non-enzymatic acetylation pathways exist, the high mitochondrial acetyl-CoA abundance facilitates spontaneous lysine acetylation [48], making deacetylation by SIRT3 the primary regulatory mechanism for maintaining energy homeostasis [49]. Beyond SIRT3, acetylation levels are also influenced by cellular metabolites, as exemplified by NAD^+^-dependent SIRT3-mediated deacetylation of aconitase [50]. In line with this regulatory role, our study showed that MLT significantly increased SIRT3 expression, reduced mitochondrial protein acetylation and enhanced ATP synthesis in spermatozoa—effects that were effectively reversed by SIRT3 inhibition with 3-TYP. Notably, although NAD^+^ is a known regulator of SIRT3 activity, no significant change was observed following MLT treatment. This may be partially attributed to the limited sample size and the fact that SIRT3 expression is also regulated by transcription factors [51]. Nevertheless, these results collectively demonstrate that SIRT3-mediated deacetylation of mitochondrial proteins is functionally linked to the enhancement of ATP synthetic capacity in boar sperm.

The regulation of energy metabolism and antioxidant activity by SIRT3 involves specific molecular targets that have been well characterized in other systems. Activating SIRT3 reduces the acetylation levels of mitochondria proteins and PDH, thus activating PDH and increasing ATP production [16]. Activation of PDH is crucial for oocytes to maintain a high ATP production rate [52]. SIRT3 overexpression reduces glycolysis metabolism in breast cancer cells by suppressing PK activity [53]. Complex I is the first and the largest component of the mitochondrial respiratory chain, and inhibition of complex I activity impairs energy metabolism and reduces ATP synthesis and sperm motility [54,55]. SIRT3 can cause hyperacetylation of complex I, and reduce ATP level [15,17]. In addition, Han et al. (2017) demonstrated that, in mouse oocytes, adding MLT to the culture medium directly stimulated SIRT3 protein expression [56]. Moreover, SIRT3 can deacetylate lysine residues on SOD2 and promote its antioxidative activity to reduce cellular oxidative stress, and these findings indicate that SOD2 is the major downstream mediator of SIRT3 in reducing cellular ROS [57]. Zhang et al. (2025) demonstrated that nicotinamide mononucleotide enhanced sperm quality parameters through stimulation of the SIRT3-SOD2/ROS pathway in pigs, which in turn results in the downregulation of SOD2 acetylation [58]. It is widely recognized that SOD is a key antioxidative enzyme: it helps boar tissues and sperm resist oxidative damage, maintain homeostasis [59,60], and is critical for cell survival and ATP production [61]. Notably, our previous study found that MLT specifically enhanced SOD activity in sperm incubated at 37 °C, with no significant changes observed in other antioxidant parameters, including total antioxidant capacity (T-AOC), reduced glutathione (GSH), and hydroxyl radical (•OH) levels [27]; this suggested that MLT primarily exerted its antioxidant effects through SOD in boar sperm. Knockout of SIRT3 resulted in a significantly increased hyperacetylation of mitochondrial proteins, accompanied by accelerated metabolic syndrome due to increased mitochondrial oxidative stress [62]. Consistent with prior reports, our data demonstrated that MLT enhanced energy metabolism and sperm motility by upregulating SIRT3 expression. This reduced mitochondrial protein acetylation, PK activity, and increased PDH expression and complex I activity. Concurrently, MLT selectively activated SOD activity, boosting antioxidant capacity, and increased ATP content. These synergistic effects were reversed by the SIRT3 inhibitor 3-TYP, indicating that MLT primarily regulates sperm ATP synthesis through SIRT3-mediated energy metabolism and antioxidant regulation. Future studies can employ techniques such as acetyl proteomics to investigate the distribution and differences in acetylated proteins in MLT-treated boar sperm, as well as their interaction with SIRT3 and ATP synthesis.

## 5. Conclusions

Our findings establish that SIRT3—a mitochondrial deacetylase activatable by MLT—functions as a pivotal metabolic regulator mediating MLT-induced enhancement of boar sperm motility. Specifically, MLT activates SIRT3 to regulate sperm energy metabolism by: (i) enhancing OXPHOS via PDH activation and respiratory complex I stimulation, and (ii) suppressing glycolysis through PK inhibition. Concurrently, SIRT3 mediates MLT-induced redox homeostasis by selectively boosting SOD activity. Collectively, these dual regulatory roles (energy metabolism + redox balance) synergistically contribute to the enhancement of metabolic flux (glucose, lactate, pyruvate, acetyl-CoA) and ATP generation, thereby augmenting MLT’s capacity to optimize sperm motility. This metabolic coordination—likely facilitated by SIRT3-dependent deacetylation—requires further verification.

While this study delineates a clear functional role for the MLT-SIRT3 axis, the proposed model of SIRT3-dependent deacetylation requires direct verification through future acetyl-proteomic studies. Furthermore, the translational potential of targeting SIRT3, demonstrated here in vitro, warrants validation in in vivo fertility trials. Notwithstanding these limitations, our work identifies SIRT3 activation as a promising therapeutic strategy to ameliorate storage-induced damage and improve boar fertility and may also provide insights for research into human male infertility.

## Figures and Tables

**Table 1:** Effect of the SIRT3 signaling pathway on sperm parameters in MLT-treated boar sperm.

Treatment	Sperm Parameters						
	Motility	Progressive Motility	Fast Motility	Circle Motility	Slow Motility	Local Motility	Immotile
Day 0	87.37 ± 0.50	78.78 ± 1.57	24.04 ± 3.66	0.34 ± 0.11	54.38 ± 3.83	8.61 ± 1.14	12.63 ± 0.50
Day 3							
VC	76.65 ± 0.13 c	64.36 ± 3.81 b	21.95 ± 6.15 b	0.37 ± 0.12 a	42.06 ± 3.19 a	12.29 ± 3.86 a	23.36 ± 0.12 a
MLT	86.77 ± 1.06 a	78.11 ± 1.75 a	40.00 ± 4.92 a	0.18 ± 0.07 a	37.92 ± 4.07 a	8.64 ± 2.47 a	13.26 ± 1.06 c
3-TYP	75.97 ± 1.27 c	62.30 ± 3.77 b	17.03 ± 3.80 b	0.32 ± 0.12 a	44.98 ± 2.74 a	13.63 ± 3.70 a	24.04 ± 1.27 a
MLT + 3-TYP	81.83 ± 0.33 b	69.66 ± 3.13 ab	22.27 ± 4.75 b	0.20 ± 0.08 a	47.20 ± 3.29 a	12.14 ± 3.26 a	18.18 ± 0.33 b

On day 3, sperm parameters were assessed using a computer-assisted sperm analysis (CASA) system following 10 min of incubation of 1 mL diluted semen aliquots at 37 °C (n = 4). Statistical significance (*p* < 0.05) between treatments (VC: 0.1% DMSO; MLT: melatonin) is indicated by different lowercase superscripts within columns.

## Data Availability

The original contributions presented in this study are included in the article/Supplementary Materials. Further inquiries can be directed to the corresponding authors.

## Supplementary Materials

The following supporting information can be downloaded at: https://www.mdpi.com/article/10.3390/cells14201633/s1, Table S1: Information on the cross-reactivity of each antibody. References [63,64,65,66,67,68,69] are cited in the Supplementary Materials File.

## Abbreviations

The following abbreviations are used in this manuscript:

ATP	Adenosine triphosphate
CASA	Computer-assisted sperm analysis system
COX IV	Cytochrome-c oxidase subunit IV
CS	Citrate synthase
DMSO	Dimethyl sulfoxide
GLUT	Glucose transporter
IDH 1/2	Isocitrate dehydrogenase
LDH	Lactate dehydrogenase
MLT	Melatonin
MMP	Mitochondria membrane potential
NAD^+^	Nicotinamide adenine dinucleotide
NADH	Reduced NAD^+^
OXPHOS	Oxidative phosphorylation
PDH	Pyruvate dehydrogenase
PK	Pyruvate kinase
SIRT3	Sirtuin 3
SOD	Superoxide dismutase
TCA	Tricarboxylic acid
VC	Vehicle control

## References

[1] Zou C.X., Yang Z.M. (2000). Evaluation on sperm quality of freshly ejaculated boar semen during in vitro storage under different temperatures. Theriogenology.

[2] Kao S.H., Chao H.T., Chen H.W., Hwang T.I., Liao T.L., Wei Y.H. (2008). Increase of oxidative stress in human sperm with lower motility. Fertil. Steril..

[3] Vyt P., Maes D., Quinten C., Rijsselaere T., Deley W., Aarts M., de Kruif A., Soom A. (2008). Detailed motility evaluation of boar semen and its predictive value for reproductive performance in sows. Vlaams Diergeneeskd. Tijdschr..

[4] Du Plessis S.S., Agarwal A., Mohanty G., van der Linde M. (2015). Oxidative phosphorylation versus glycolysis: What fuel do spermatozoa use?. Asian J. Androl..

[5] Marin S., Chiang K., Bassilian S., Lee W.-N.P., Boros L.G., Fernández-Novell J.M., Centelles J.J., Medrano A., Rodriguez-Gil J.E., Cascante M. (2003). Metabolic strategy of boar spermatozoa revealed by a metabolomic characterization. FEBS Lett..

[6] Nevo A.C., Polge C., Frederick G. (1970). Aerobic and anaerobic metabolism of boar spermatozoa in relation to their motility. Reproduction.

[7] Nakada K., Sato A., Yoshida K., Morita T., Tanaka H., Inoue S.-I., Yonekawa H., Hayashi J.-I. (2006). Mitochondria-related male infertility. Proc. Natl. Acad. Sci. USA.

[8] John J.C.S., John B.S., Zini A., Agarwal A. (2011). Sperm mitochondrial DNA. Sperm Chromatin: Biological and Clinical Applications in Male Infertility and Assisted Reproduction.

[9] Pfeiffer T., Schuster S., Bonhoeffer S. (2001). Cooperation and competition in the evolution of ATP-producing pathways. Science.

[10] Jane Rogers B., Yanagimachi R. (1975). Retardation of guinea pig sperm acrosome reaction by glucose: The possible importance of pyruvate and lactate metabolism in capacitation and the acrosome reaction. Biol. Reprod..

[11] Dziekońska A., Fraser L., Strzeżek J. (2009). Effect of different storage temperatures on the metabolic activity of spermatozoa following liquid storage of boar semen. J. Anim. Feed Sci..

[12] Amaral A. (2022). Energy metabolism in mammalian sperm motility. WIREs Mech. Dis..

[13] Piomboni P., Focarelli R., Stendardi A., Ferramosca A., Zara V. (2012). The role of mitochondria in energy production for human sperm motility. Int. J. Androl..

[14] Lantier L., Williams A.S., Williams I.M., Yang K.K., Bracy D.P., Goelzer M., James F.D., Gius D., Wasserman D.H. (2015). SIRT3 is crucial for maintaining skeletal muscle insulin action and protects against severe insulin resistance in high-fat–fed mice. Diabetes.

[15] Ahn B.-H., Kim H.-S., Song S., Lee I.H., Liu J., Vassilopoulos A., Deng C.-X., Finkel T. (2008). A role for the mitochondrial deacetylase Sirt3 in regulating energy homeostasis. Proc. Natl. Acad. Sci. USA.

[16] Sun Y., Tian Z., Liu N., Zhang L., Gao Z., Sun X., Yu M., Wu J., Yang F., Zhao Y. (2018). Exogenous H_2_S switches cardiac energy substrate metabolism by regulating SIRT3 expression in db/db mice. J. Mol. Med..

[17] Lombard D.B., Alt F.W., Cheng H.-L., Bunkenborg J., Streeper R.S., Mostoslavsky R., Kim J., Yancopoulos G., Valenzuela D., Murphy A. (2007). Mammalian Sir2 Homolog SIRT3 Regulates Global Mitochondrial Lysine Acetylation. Mol. Cell. Biol..

[18] Zhang H., Chai J., Cao C., Wang X., Pang W. (2024). Supplementing Boar Diet with Nicotinamide Mononucleotide Improves Sperm Quality Probably through the Activation of the SIRT3 Signaling Pathway. Antioxidants.

[19] Gholami M., Saki G., Hemadi M., Khodadadi A., Mohammadi-Asl J. (2014). Melatonin improves spermatogonial stem cells transplantation efficiency in azoospermic mice. Iran. J. Basic. Med. Sci..

[20] Barranco I., Casao A., Perez-Patiño C., Parrilla I., Muiño-Blanco T., Martinez E.A., Cebrian-Perez J.A., Roca J. (2017). Profile and reproductive roles of seminal plasma melatonin of boar ejaculates used in artificial insemination programs1. J. Anim. Sci..

[21] Yang D., Wang C., Lu W., Tian X., Sun Y., Peng H. (2024). Beneficial effects of melatonin on boar sperm motility and kinematics are mediated by MT1 receptor. Theriogenology.

[22] Pezo F., Zambrano F., Uribe P., Moya C., de Andrade A.F.C., Risopatron J., Yeste M., Burgos R.A., Sanchez R. (2021). Oxidative and nitrosative stress in frozen-thawed pig spermatozoa. I: Protective effect of melatonin and butylhydroxytoluene on sperm function. Res. Vet. Sci..

[23] Ofosu J., Qazi I.H., Fang Y., Zhou G. (2021). Use of melatonin in sperm cryopreservation of farm animals: A brief review. Anim. Reprod. Sci..

[24] Suofu Y., Li W., Jean-Alphonse F.G., Jia J., Khattar N.K., Li J., Baranov S.V., Leronni D., Mihalik A.C., He Y. (2017). Dual role of mitochondria in producing melatonin and driving GPCR signaling to block cytochrome c release. Proc. Natl. Acad. Sci. USA.

[25] Owino S., Buonfiglio D.D.C., Tchio C., Tosini G. (2019). Melatonin Signaling a Key Regulator of Glucose Homeostasis and Energy Metabolism. Front. Endocrinol..

[26] Deng C.-C., Zhang J.-P., Huo Y.-N., Xue H.-Y., Wang W., Zhang J.-J., Wang X.-Z. (2022). Melatonin alleviates the heat stress-induced impairment of Sertoli cells by reprogramming glucose metabolism. J. Pineal Res..

[27] Lu N., Jiang X., Zhang C., Li B., Tu W., Lei H., Yao W., Xia D. (2022). Melatonin mediates via melatonin receptor 1 in a temperature-dependent manner regulating ATP metabolism and antioxidative enzyme activity of boar spermatozoa in vitro. Theriogenology.

[28] Pi H., Xu S., Reiter R.J., Guo P., Zhang L., Li Y., Li M., Cao Z., Tian L., Xie J. (2015). SIRT3-SOD2-mROS-dependent autophagy in cadmium-induced hepatotoxicity and salvage by melatonin. Autophagy.

[29] Gong Y., Guo H., Zhang Z., Zhou H., Zhao R., He B. (2017). Heat Stress Reduces Sperm Motility via Activation of Glycogen Synthase Kinase-3α and Inhibition of Mitochondrial Protein Import. Front. Physiol..

[30] Chen Y., Liang P., Huang Y., Li M., Zhang X., Ding C., Feng J., Zhang Z., Zhang X., Gao Y. (2017). Glycerol kinase-like proteins cooperate with Pld6 in regulating sperm mitochondrial sheath formation and male fertility. Cell Discov..

[31] Ozaki Y., Ohashi K., Otaka N., Kawanishi H., Takikawa T., Fang L., Takahara K., Tatsumi M., Ishihama S., Takefuji M. (2023). Myonectin protects against skeletal muscle dysfunction in male mice through activation of AMPK/PGC1α pathway. Nat. Commun..

[32] Brydon L., Petit L., Delagrange P., Strosberg A.D., Jockers R. (2001). Functional Expression of MT2 (Mel1b) Melatonin Receptors in Human PAZ6 Adipocytes. Endocrinology.

[33] Rocha C.S., Martins A.D., Rato L., Silva B.M., Oliveira P.F., Alves M.G. (2014). Melatonin alters the glycolytic profile of Sertoli cells: Implications for male fertility. Mol. Hum. Reprod..

[34] Fang Y., Zhao C., Xiang H., Zhao X., Zhong R. (2020). Melatonin Inhibits Formation of Mitochondrial Permeability Transition Pores and Improves Oxidative Phosphorylation of Frozen-Thawed Ram Sperm. Front. Endocrinol..

[35] Medrano A., Fernández-Novell J.M., Ramió L., Alvarez J., Goldberg E., Montserrat Rivera M., Guinovart J.J., Rigau T., Rodríguez-Gil J.E. (2006). Utilization of citrate and lactate through a lactate dehydrogenase and ATP-regulated pathway in boar spermatozoa. Mol. Reprod. Dev..

[36] Cai X., Ng C.P., Jones O., Fung T.S., Ryu K.W., Li D., Thompson C.B. (2023). Lactate activates the mitochondrial electron transport chain independently of its metabolism. Mol. Cell.

[37] Jackson L.E., Kulkarni S., Wang H., Lu J., Prochownik E.V. (2022). Genetic Dissociation of Glycolysis and the TCA Cycle Affects Neither Normal nor Neoplastic Proliferation. Cancer Res..

[38] Gray L.R., Tompkins S.C., Taylor E.B. (2014). Regulation of pyruvate metabolism and human disease. Cell. Mol. Life Sci..

[39] Prieto O.B., Algieri C., Spinaci M., Trombetti F., Nesci S., Bucci D. (2023). Cell bioenergetics and ATP production of boar spermatozoa. Theriogenology.

[40] Brooks D.E., Mann T. (1973). Pyruvate Metabolism in Boar Spermatozoa. Reproduction.

[41] Hereng T.H., Elgstøen K.B.P., Cederkvist F.H., Eide L., Jahnsen T., Skålhegg B.S., Rosendal K.R. (2011). Exogenous pyruvate accelerates glycolysis and promotes capacitation in human spermatozoa. Hum. Reprod..

[42] Kumar N. (2023). Sperm mitochondria, the driving force behind human spermatozoa activities: Its functions and dysfunctions-a narrative review. Curr. Mol. Med..

[43] Costa J., Braga P.C., Rebelo I., Oliveira P.F., Alves M.G. (2023). Mitochondria quality control and male fertility. Biology.

[44] Wang Q., Zhang Y., Yang C., Xiong H., Lin Y., Yao J., Li H., Xie L., Zhao W., Yao Y. (2010). Acetylation of metabolic enzymes coordinates carbon source utilization and metabolic flux. Science.

[45] Yang X., Seto E. (2008). Lysine acetylation: Codified crosstalk with other posttranslational modifications. Mol. Cell.

[46] Anderson K.A., Hirschey M.D. (2012). Mitochondrial protein acetylation regulates metabolism. Essays Biochem..

[47] Kim S.C., Sprung R., Chen Y., Xu Y., Ball H., Pei J., Cheng T., Kho Y., Xiao H., Xiao L. (2006). Substrate and functional diversity of lysine acetylation revealed by a proteomics survey. Mol. Cell.

[48] Baeza J., Smallegan M.J., Denu J.M. (2015). Site-Specific Reactivity of Nonenzymatic Lysine Acetylation. Acs Chem. Biol..

[49] Newman J.C., He W., Verdin E. (2012). Mitochondrial Protein Acylation and Intermediary Metabolism: Regulation by Sirtuins and Implications for Metabolic Disease. J. Biol. Chem..

[50] Fernandes J., Weddle A., Kinter C.S., Humphries K.M., Kinter M. (2015). Lysine Acetylation Activates Mitochondrial Aconitase in the Heart. Biochemistry.

[51] Song C., Fu B., Zhang J., Zhao J., Yuan M., Peng W., Zhang Y., Wu H. (2018). Sodium fluoride induces nephrotoxicity via oxidative stress-regulated mitochondrial SIRT3 signaling pathway. Sci. Rep..

[52] Imanaka S., Shigetomi H., Kobayashi H. (2022). Reprogramming of glucose metabolism of cumulus cells and oocytes and its therapeutic significance. Reprod. Sci..

[53] Zu Y., Chen X.-F., Li Q., Zhang S.-T., Si L.-N. (2021). PGC-1α activates SIRT3 to modulate cell proliferation and glycolytic metabolism in breast cancer. Neoplasma.

[54] Lannuzel A., Michel P.P., Höglinger G.U., Champy P., Jousset A., Medja F., Lombès A., Darios F., Gleye C., Laurens A. (2003). The mitochondrial complex I inhibitor annonacin is toxic to mesencephalic dopaminergic neurons by impairment of energy metabolism. Neuroscience.

[55] Plaza Davila M., Martin Muñoz P., Tapia J.A., Ortega Ferrusola C., Balao da Silva C.C., Peña F.J. (2015). Inhibition of Mitochondrial Complex I Leads to Decreased Motility and Membrane Integrity Related to Increased Hydrogen Peroxide and Reduced ATP Production, while the Inhibition of Glycolysis Has Less Impact on Sperm Motility. PLoS ONE.

[56] Han L., Wang H., Li L., Li X., Ge J., Reiter R.J., Wang Q. (2017). Melatonin protects against maternal obesity-associated oxidative stress and meiotic defects in oocytes via the SIRT 3-SOD 2-dependent pathway. J. Pineal Res..

[57] Qiu X., Brown K., Hirschey M.D., Verdin E., Chen D. (2010). Calorie Restriction Reduces Oxidative Stress by SIRT3-Mediated SOD2 Activation. Cell Metab..

[58] Zhang H., Qin X., Bojan N., Cao C., Chai J., Pang W. (2025). Nicotinamide mononucleotide enhances porcine sperm quality by activating the SIRT3-SOD2/ROS pathway and promoting oxidative phosphorylation. Anim. Reprod. Sci..

[59] Shamsi M.B., Venkatesh S., Tanwar M., Talwar P., Sharma R.K., Dhawan A., Kumar R., Gupta N.P., Malhotra N., Singh N. (2009). DNA integrity and semen quality in men with low seminal antioxidant levels. Mutat. Res..

[60] Koziorowska-Gilun M., Koziorowski M., Fraser L., Strzeżek J. (2011). Antioxidant defence system of boar cauda epididymidal spermatozoa and reproductive tract fluids. Reprod. Domest. Anim..

[61] Zhu Y., Park S.-H., Ozden O., Kim H.-S., Jiang H., Vassilopoulos A., Spitz D.R., Gius D. (2012). Exploring the electrostatic repulsion model in the role of Sirt3 in directing MnSOD acetylation status and enzymatic activity. Free Radic. Biol. Med..

[62] Hirschey M.D., Shimazu T., Goetzman E., Jing E., Schwer B., Lombard D.B., Grueter C.A., Harris C., Biddinger S., Ilkayeva O.R. (2010). SIRT3 regulates mitochondrial fatty-acid oxidation by reversible enzyme deacetylation. Nature.

[63] Zhang L., Wang Z., Zhang J., Luo X., Du Q., Chang L., Zhao X., Huang Y., Tong D. (2018). Porcine parvovirus infection impairs progesterone production in luteal cells through mitogen-activated protein kinases, p53, and mitochondria-mediated apoptosis. Biol. Reprod..

[64] Brownstein A.J., Ganesan S., Summers C.M., Pearce S., Hale B.J., Ross J.W., Gabler N., Seibert J.T., Rhoads R.P., Baumgard L.H. (2017). Heat stress causes dysfunctional autophagy in oxidative skeletal muscle. Physiol. Rep..

[65] Li Y., Xu S., Jiang B., Cohen R.A., Zang M. (2013). Activation of sterol regulatory element binding protein and NLRP3 inflammasome in atherosclerotic lesion development in diabetic pigs. PLoS One.

[66] Liu L., Lu H., Loor J.J., Aboragah A., Du X., He J., Peng T., Su J., Wang Z., Liu G. (2021). Sirtuin 3 inhibits nuclear factor-κB signaling activated by a fatty acid challenge in bovine mammary epithelial cells. J. Dairy Sci..

[67] Liu L., Xing D., Du X., Peng T., McFadden J.W., Wen L., Lei H., Dong W., Liu G., Wang Z. (2020). Sirtuin 3 improves fatty acid metabolism in response to high nonesterified fatty acids in calf hepatocytes by modulating gene expression. J. Dairy Sci..

[68] Sun J., Su F., Chen Y., Wang T., Ali W., Jin H., Xiong L., Ma Y., Liu Z., Zou H. (2024). Co-exposure to PVC microplastics and cadmium induces oxidative stress and fibrosis in duck pancreas. Sci. Total Environ..

[69] Wang J., Quan R., He X., Fu Q., Tian S., Zhao L., Li S., Shi L., Li R., Chen B. (2023). Hypovirus infection induces proliferation and perturbs functions of mitochondria in the chestnut blight fungus. Front. Microbiol..

