# Supplementary material for: SIRT3 Mediates Coordination Between Energy Metabolism and SOD Activity in Melatonin-Enhanced Boar Sperm Motility
Source: Cells. 2025 Oct 20;14(20):1633. doi: 10.3390/cells14201633 (PMC12564921; doi:10.3390/cells14201633)
Supplement: Supplementary file 1 [file cells-14-01633-s001.zip › cells-3898690-supplementary.pdf]

Table S1. Information on the cross-reactivity of each antibody.

| Protein symbol | Product No. | Source | Cross reactivity |
|---|---|---|---|
| COX IV | sc-376731 | Mouse | Human (Homology with pigs: 81.07%) |
| SIRT3 | sc-365175 | Mouse | Mouse, Rat, Human (Homology with pigs: 80.66%) |
| acetylated lysine | 9441s | Rabbit | All |
| PDH | 3205s | Rabbit | Human, Mouse, Rat, Monkey (Homology with pigs: 97.68%) |
| CS | Bs-17709R | Rabbit | Human (predicted: Mouse, Rat, Cow, Zebrafish, Dog) (Homology with pigs: 96.12%) |
| IDH 1/2 | sc-373816 | Mouse | Mouse, Rat, Human, Equine, Canine, Bovine, **Porcine**, Avian (Homology with pigs: 96.68%) |

Notes: The COX IV antibody was used to tested in porcine steroidogenic luteal cells [1], the PDH antibody was used to tested in porcine skeletal muscle [2], and the acetylated lysine antibody was used to tested in porcine aortic tissue [3]. In addition, the SIRT3 antibody had been successfully tested in bovine mammary epithelial cells [4], calf hepatocytes [5] (Homology with bovine: 76.47%) and in duck pancreas [6] (Homology with duck: 74.67%), the CS antibody had been successfully tested in chestnut blight fungus (bacteria) [7].

## References:


1. Zhang, L.; Wang, Z.; Zhang, J.; Luo, X.; Du, Q.; Chang, L.; Zhao, X.; Huang, Y.; Tong, D. Porcine parvovirus infection impairs progesterone production in luteal cells through mitogen-activated protein kinases, p53, and mitochondria-mediated apoptosis. *Biol. Reprod.* **2018**, *98*, 558-569.
2. Brownstein, A.J.; Ganesan, S.; Summers, C.M.; Pearce, S.; Hale, B.J.; Ross, J.W.; Gabler, N.; Seibert, J.T.; Rhoads, R.P.; Baumgard, L.H.; et al. Heat stress causes dysfunctional autophagy in oxidative skeletal muscle. *Physiol. Rep.* **2017**, *5*, e13317.
3. Li, Y.; Xu, S.; Jiang, B.; Cohen, R.A.; Zang, M. Activation of sterol regulatory element binding protein and NLRP3 inflammasome in atherosclerotic lesion development in diabetic pigs. *PLoS One* **2013**, *8*, e67532.
4. Liu, L.; Lu, H.; Loor, J.J.; Aboragah, A.; Du, X.; He, J.; Peng, T.; Su, J.; Wang, Z.; Liu, G.; et al. Sirtuin 3 inhibits nuclear factor-κB signaling activated by a fatty acid challenge in bovine mammary epithelial cells. *J. Dairy Sci.* **2021**, *104*, 12871-12880.
5. Liu, L.; Xing, D.; Du, X.; Peng, T.; McFadden, J.W.; Wen, L.; Lei, H.; Dong, W.; Liu, G.; Wang, Z.; et al. Sirtuin 3 improves fatty acid metabolism in response to high nonesterified fatty acids in calf hepatocytes by modulating gene expression. *J. Dairy Sci.* **2020**, *103*, 6557-6568.
6. Sun, J.; Su, F.; Chen, Y.; Wang, T.; Ali, W.; Jin, H.; Xiong, L.; Ma, Y.; Liu, Z.; Zou, H. Co-exposure to PVC microplastics and cadmium induces oxidative stress and fibrosis in duck pancreas. *Sci. Total Environ.* **2024**, *927*, 172395.
7. Wang, J.; Quan, R.; He, X.; Fu, Q.; Tian, S.; Zhao, L.; Li, S.; Shi, L.; Li, R.; Chen, B. Hypovirus infection induces proliferation and perturbs functions of mitochondria in the chestnut blight fungus. *Front. Microbiol.* **2023**, *14*, 1206603.
